# The effect of *Toxoplasma gondii* on plasma serotonin concentration in sheep

**DOI:** 10.14202/vetworld.2018.1500-1505

**Published:** 2018-10-26

**Authors:** Annamaria Castello, Giuseppe Bruschetta, Renato Paolo Giunta, Anna Maria Fausta Marino, Alida Maria Ferlazzo

**Affiliations:** 1 Italian National Reference Center for Toxoplasmosis (Ce.Tox) - Experimental Zooprophylactic Institute of Sicily (IZS), Via Passo Gravina 195, 95125 Catania, Italy; 2 Department of Veterinary Sciences, University of Messina, Polo Universitario dell’Annunziata, 98168 Messina, Italy

**Keywords:** monocytes, plasma, serotonin, serum, sheep, toxoplasmosis

## Abstract

**Background and Aim:**

*Toxoplasma gondii* is an intracellular parasite that commonly infects warm-blooded animals, including humans. Virtually all species can be infected, but a species-specific variability is evident, in terms of both type and severity of the symptoms encountered. As serotonin (5-hydroxytryptamine [5-HT]) plays an important regulatory role in both physiological and immune responses, the aim of this research was to assess whether toxoplasmosis disease could affect plasma 5-HT concentration and/or hematochemical parameters in a particularly susceptible species to infection as sheep.

**Materials and Methods:**

5-HT plasma levels were analyzed in platelet-poor plasma fraction by enzyme-linked immunosorbent assay. Blood count and hematochemical parameters were evaluated. Total proteins (TPs), glucose (Glu), and lactate dehydrogenase were determined by a spectrophotometer.

**Results:**

Results showed significantly higher levels in plasma 5-HT, monocytes, and TP and significantly lower levels of Glu, in infected sheep compared to the control group.

**Conclusion:**

Results could support the hypothesis of an effect of toxoplasmosis infection on plasma 5-HT concentrations in sheep. More research is needed to assess the function of 5-HT in the regulation of infected sheep’s immune responses.

## Introduction

*Toxoplasma gondii* is an intracellular parasite that commonly infects warm-blooded animals, including humans. Usually, *T. gondii* infection runs asymptomatically or causes mild clinical signs in immunocompetent, non-pregnant individuals. Among domesticated animals, small ruminants and swine are the most often infected species; they show a wide variety of non-specific symptoms, ranging from fever and dyspnea among the first ones and fever, depression, lethargy, vomiting, diarrhea, chorioretinitis, and lymphadenopathy among the latter [1] (http://www.cdc.gov/ncidod/dpd/parasites/toxoplasmosis/default.htm). In both species, the most severe consequences of this parasitic infection arise from primary *T. gondii* infection during early pregnancy and subsequent transplacental transmission to the fetus. In this case, mild to serious symptoms can be observed, such as stillbirth, miscarriage, mummification, resorption of the fetus, or congenital toxoplasmosis signs against the newborn, including central nervous system involvement, retinochoroiditis, and subclinical infection at birth with a risk of late onset of ocular diseases.

In addition to the well-known previously mentioned symptoms, recently several studies have highlighted or hypothesized a correlation between latent toxoplasmosis and various neuropsychiatric disorders [2,3] and an alteration in neurotransmission has been suggested as one of the factors influencing part of these mood disorders [4].

Even though it is evident that different species can show different reactions to *T. gondii* infection, in terms of both type and severity of the symptoms, most of the studies are usually performed on mice [5], while a deeper comprehension of host’s physiological and immune mechanisms activated by this parasite in other species might be interesting not only for veterinary but also for human medicine. In fact, on the one side, these evaluations might contribute to better understand which factors influence the different level of susceptibility observed among the different animal species, while on the other one side, they might be traced to humans and help to elaborate hypothesis about physiological and immune mechanisms underlying the evolution of toxoplasmosis symptoms in humans.

Basing on the aforementioned considerations, serotonin or 5-hydroxytryptamine (5-HT) might be of particular interest. In fact, it shows both hormonal and immune-modulatory functions. 5-HT is synthesized from the amino acid tryptophan in two-step reactions. The first one, rate limiting, is independently activated in peripheral tissues and brain by two different tryptophan hydroxylase (TPH) (TPH-1 and TPH-2) isoforms. The TPH-1 gene is mainly expressed in the gut and controls the production of 5-HT in the periphery. 5-HT is primarily produced in the enterochromaffin cells of the intestine [6]. Circulating levels of 5-HT are extremely low, as the main amount is stored in platelets, which, in contrast to neurons, cannot synthesize 5-HT but are its main peripheral uptake system by means of the 5-HT serotonin transporter [7].

Different studies reach seemingly opposite conclusions on the role played in immune responses by 5-HT, which show in some cases pro-inflammatory functions [8] and others anti-inflammatory ones [9]. 5-HT could be involved in the modulation of all cellular lines of the immune system, though its effects seem to depend on its concentration and the class of receptor-activated [10-12]. 5-HT-dependent mechanisms have been described for dendritic cells and neutrophils recruitment, leukocytes recruitment, and adhesion, as well as for the release of cytokines at the inflammation site. T lymphocytes express numerous 5-HT receptors; B lymphocytes, under the effect of a serotoninergic system stimulation, undergo proliferation; and also natural killer (NK) cell activity is modulated by 5-HT [13]. Based on these observations, it is clear how 5-HT can be a promising target for the modulation of immune responses, as well as a factor involved in many pathological alterations. A correlation between nervous system impairment caused by *T. gondii* and 5-HT alterations can be hypothesized but, while contradicting data are available on 5-HT levels at target tissues, depending on both the examined tissue and the breed, strain, or sex of the animals used in previous studies [5,14,15], no data on 5-HT plasma levels were found.

The aim of this research was to assess the variation of plasma 5-HT concentration among infected and uninfected sheep (control group), a particularly susceptible species to *T. gondii* infection, to assess whether a variation of blood 5-HT concentration and/or hematochemical parameters might be related to the infection. Such analyses were carried out to understand if 5-HT might be considered a relevant indicator of sheep immune responses to *T. gondii* and to better clarify the physiological bases of the different host susceptibility to *T. gondii*.

## Materials and Methods

### Ethical approval

All methods and procedures used in this study were approved by the Animal Care and Use Committee of University of Messina, in accordance with EU Directive 2010/63/EU for animal experiments.

### Animals

The study was carried out on 57 female sheep, heterogeneous breed, sampled from three farms in the area of Catania. There was a history of abortion due to *T. gondii* in the past but not during the investigation. Furthermore, none of the selected sheep was pregnant. Each animal was given a progressive identification number from 1 to 57. All sheep were reared under similar conditions, contemplating grazing during the morning and night shelter. Animals were maintained at the same food regime, grazing partly of wild herbs of the area and partly of arable ones, including the same fodder integration during periods of non-arable pasture. Animals were accustomed to individual housing, during a previous period of 4 months. Sheep were about 3±2 years and weighed 45±15 kg. The number of animals sampled in each farm is listed in Table-1, each farm is referred to as A, B, and C.

**Table-1:** Number of animals sampled in each farm.

Farm	Number of sampled animals
A	15
B	9
C	33

### Sample collection

Blood samples were collected from a jugular vein between 9:00 am and 11:00 am, in June and October. All samples were taken by the same operator. Two aliquots of blood were collected from each animal, one was put in a tube for serum isolation (evacuated tubes; Z serum clot activator, Vacuette^®^, Greiner Bio-one, Kremsmünster, Austria) and another one was put in tubes with EDTA (evacuated tubes, K3-EDTA, Vacuette^®^, Greiner Bio-one, Kremsmünster, Austria), used to evaluate hematological parameters and to isolate plasma. Both groups of tubes were immediately refrigerated at 4°C.

### Determination of positivity or negativity to infection by T. gondii

Positivity or negativity to *T. gondii* infection was determined by application of the protocol “POS-CE. TOX01: Serological diagnosis of toxoplasmosis by enzyme-linked immunosorbent assay (ELISA) in ovine and caprine animals serum (PriocheckAb SR),” validated and accredited (ACCREDIA) by the National Reference Centre for Toxoplasmosis (Ce.Tox)-Catania, Italy. The ELISA Assay kit used is PrioCHECK^®^ Toxoplasma Ab SR, Prionics AG, Schlieren-Zurich, Switzerland. Optical densities (ODs) were measured on two wells per sample at λ=450 nm, using the microplate reader BIO-RAD 680, BIO-RAD Laboratories, Segrate, Italy. The percentage of positivity (PP) of samples and weakly positive control, required to validate the test, was calculated according to the following formula:


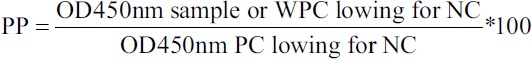


PC = Positive control provided by the kit, average of two OD values

WPC = Weakly positive control provided by the kit, average of two OD values

NC = Negative control provided by the kit, average of two OD values.

The test validation criteria, required to accept the results, are listed below:

Average of PC OD450 ≥1.2

WPC PP ≥35%

Average of NC OD450 < 0.15.

Samples were considered positive to *T. gondii* infection if their PP value was >20%, while samples whose PP was <20% were considered uninfected and used as a control group. On the basis of the approach described, 17 animals were tested positive for *T. gondii* infection, and 40 animals were tested uninfected by the parasite (Table-2).

**Table-2:** Number of infected and uninfected animals sampled in each farm.

Farm	Number of sampled animals		

	Infected by *T. gondii*	Uninfected	Total
A	7	8	15
B	2	7	9
C	8	25	33

T. gondii=Toxoplasma gondii

### Blood count and hematochemical parameter evaluation

Hematocrit %, red blood cells, mean cell volume, mean cell hemoglobin (MCH), MCH concentration, white blood cells, neutrophils, lymphocytes, monocytes, eosinophils, basophils, and platelets were measured using the automatic counting machine Cell-Dyn 3700, GMI Company, Ramsey, Minnesota, USA.

The concentration of total proteins (TPs) and glucose (Glu) and the activity of lactate dehydrogenase were determined in sera using the automatic analyzer Konelab 60 (Thermo Electron Corporation, Waltham, Massachusetts, USA). The biochemical and hematological parameters determined are listed in Table-3.

**Table-3:** Biochemical and hematological parameters (mean±SD) measured in infected and uninfected animals.

Biochemical/hematological parameters	Infected animals	Uninfected animals
Hemoglobin (g/dl)	11.19±1.54	10.45±0.83
HCT, %	33.5±4.5	31.6±2.5
RBC (×10^6^/ml)	10.159±0.998	9.981±0.827
MCV (fl)	32.98±2.45	31.98±2.73
MCH (pg)	10.98±0.99	10.51±0.87
MCHC (g/dl)	32.76±0.87	33.25±1.09
WBC (×10^3^/ml)	8.232±2.170	8.245±1.570
Neutrophils (×10^3^/ml)	3.404±1.394	2.844±1.071
Lymphocytes (×10^3^/ml)	4.201±1.708	4.552±1.850
Monocytes (×10^3^/ml)	0.461±0.332	0.188±0.130^b^
Eosinophils (×10^3^/ml)	0.523±0.334	0.553±0.212
Basophils (×10^3^/ml)	0.036±0.016	0.029±0.017
Platelets (×10^5^/ml)	5.65±1.17	5.73±1.48
TP (g/dl)	8.00±0.78	7.36±0.44^b^
Glu (mg/dl)	39.5±7.7	46.0±7.7^a^
LDH (U/l)	433.3±110.0	428.8±47.6

Letters indicate significant differences versus infected animals: ^a^p<0.01; ^b^p<0.001. LDH=Lactate dehydrogenase, SD=Standard deviation, HCT=Hematocrit, RBC=Red blood cells, MCV=Mean cell volume, MCHC=Mean cell hemoglobin concentration, WBC=White blood cells, TP=Total proteins, Glu=Glucose

### Analysis of plasma 5-HT concentration

**Figure-1:**
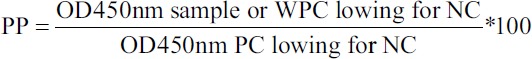
Plasma concentrations of serotonin or 5-hydroxytryptamine (mean±standard deviation) in sheep. Letter indicates significant differences between uninfected and infected animals: ^a^p<0.001.

5-HT concentration was measured in platelet-poor plasma fraction using the ELISA Assay kit “DLD Serotonin ELISA” (Ref. EA602/96), DLD Diagnostika GmbH, Hamburg, Germany. For each ELISA test performed, the standards provided by the kit were also processed, according to the reference protocol. OD values obtained for the standards were used for the extrapolation of the reference semi-logarithmic curve as in the above-mentioned protocol. The concentration of 5-HT contained in each plasma sample was obtained returning the OD value measured in the corresponding well on the curve and dividing the read-off value by a factor of 1.8. The mean and standard deviation (SD) values calculated for infected sheep and control group are reported in the histogram in Figure-1.

### Statistical analysis

All results are expressed as mean values±SD. Statistical analysis was performed by application of Student’s t-test for unpaired data. The level of significance was set at p<0.05. All calculations were performed using the PRISM software (GraphPad Software Inc., San Diego, CA, USA).

## Results

Among the 57 sheep included in this analysis, 17 animals were positive for *T. gondii*, and 40 animals were uninfected. To the best of our knowledge, seropositivity does not prove clinical toxoplasmosis. For our study, we selected the subjects that had seroconverted in the previous 4 months to investigate animals that were recently exposed/infected with *T. gondii*. The serological screening was carried out using a validated method complying with the requirements of the OIE manual. A number of infected and uninfected animals sampled in each farm are listed in Table-2.

*T. gondii*-infected sheep had significantly higher levels of monocytes (p<0.001) and TPs (p<0.001) and significantly lower levels of Glu (p<0.01) in comparison to uninfected animals (Table-3). Moreover, the group of infected animals had a significantly higher level of plasma 5-HT (p<0.001) compared to control group (Figure-1). The same result was obtained also separating samples basing on age range and sampling season.

## Discussion

Toxoplasmosis is a particularly widespread parasitic zoonosis both in wild animals and domestic species. Although the relevance of such parasitism, not only in the veterinary field but also in humans, is clear from the 1940s and even though it is among the most popular food-borne zoonoses in Europe [16], the extent of the public health problem is still underestimated. The reason for this underrating of the phenomenon is mainly due to the absence of symptoms found in most immunocompetent hosts contracting the infection. When symptoms are observed, they may be particularly variable in both typology and severity, depending on the species of infected hosts. Given the variability of the symptoms observed among the host species, the purpose of the present study is to evaluate some general hematochemical parameters as well as hormonal markers hypothetically related to the responses to such parasitosis, in positive and negative animals belonging to the aforementioned species. 5-HT was proved as a key regulator of many cells of the immune system; therefore, its variation may represent an interesting factor to consider in correlation to toxoplasmosis. Given that the effect of *T. gondii* cyst formation in the brain apparently causes a decrease of 5-HT production in this site, it is still unknown whether the host’s contact with the parasite causes the same hormonal alteration at circulating blood level, observations that could lead to interesting considerations regarding the immunomodulatory role played by 5-HT in response to *T. gondii* infections. From the analysis performed, a significant difference among the two groups in the white line cellular component, known to respond to infection, was highlighted. In particular, positive animals to toxoplasmosis exhibited higher levels of monocytes. A recent study [17] proposed that the enhanced motility of infected monocytes supports dissemination of the parasite during acute infection and this reprogramming of monocyte motility contributes to *T. gondii* survival in mammalian hosts. Moreover, infected animals showed a significant increase in TP and a significant decrease in blood Glu levels compared to the control group. The last data would be of particular interest since, according to the bibliography, the guest Glu would be useful for the growth and multiplication of *T. gondii*, although not indispensable [18]. In fact, under Glu starvation, the parasite can still multiply, using other metabolites as energy sources as glutamine and acetate. Mutual cooperation of these metabolites is essential for the lytic cycle of the obligate intracellular parasite *T. gondii*, to ensure the biomass, energy, and redox demands during all proliferation as well as differentiation [19].

Moreover, *T. gondii* can tolerate the deletion of its surface Glu transporter (*T. gondii* glucose transporter), and it thrives by catabolizing glutamine to sustain its metabolism [18]. These data mean that Glu is not the only substratum that can be used by the parasite but also certainly the first one to be exploited and the most efficient energy source. Considering that sheep is a species particularly susceptible to *T. gondii* infection and that in infected animals, blood Glu is lower than that in uninfected ones, it might be interesting to make a wider comparison, including some other susceptible species and some less susceptible ones, to understand if this variation can be a common element among susceptible species and at the same time a discriminating factor against the least susceptible ones. A significant increase of TP levels observed in infected compared to uninfected sheep can be justified, as already observed in young humans [20], by the dehydration related to infection. It is known that in young or immunosuppressed people, affected by *T. gondii* infection, enteric symptoms such as diarrhea [21], dehydration, weight loss, and debilitation [22] can be observed.

Regarding the evaluation of circulating 5-HT levels, significantly higher levels of 5-HT in infected sheep compared to the control group were observed. It is well known that 5-HT plays a role in immune responses, which in some cases include pro-inflammatory [8] and in others anti-inflammatory functions [9]. The mechanisms underlying one or other function are still unclear, although it is well known that the occurrence of the first or second condition also depends on the type of receptors activated or inhibited [10,11] by the neurotransmitter and how 5-HT levels divert from physiological concentrations. Arzt *et al*. [23] and Cloëz-Tayarani *et al*. [10] showed that 5-HT, in a broad spectrum of concentrations, inhibited lipopolysaccharide-induced tumor necrosis factor α (TNFα) synthesis. Moreover, it has been demonstrated that 5-HT in physiological concentrations may increase interleukin-6 and TNFα production partly by stimulation of the 5-HT_2_ receptors and that extracellular 5-HT concentration above the baseline physiological levels may suppress the production of the above cytokines [24].

5-HT is synthesized and released in the bloodstream by the enterochromaffin cells of gastric and intestinal mucosa [6]. The main amounts of 5-HT are rapidly taken up by platelets, where it is stored at high concentrations, and to a lesser extent by lymphocytes and monocytes [25]. In inflammatory conditions, the activated platelets release 5-HT, and this leads to an increase in its local concentration at the inflamed region. 5-HT interacts with blood cells, and its modulatory effect has been well documented in innate as well as adaptive immunity. It stimulates monocytes and lymphocytes and hence influences the secretion of cytokines. Particularly, 5-HT function is known for T lymphocytes, B lymphocytes, NK cells, and monocytes/macrophages [13]. At inflammatory sites, also bacteria or parasites activate platelets, resulting in 5-HT secretion [26]. The results obtained in this study, which showed a significant parallel increase of both plasma 5-HT and monocyte concentrations, seem to be in line with the already known important immunoregulatory function of 5-HT, even though the complexity of peripheral 5-HT effects does not allow to draw definitive conclusions. Nevertheless, as 5-HT seems to be a promising new target to control immune responses, it would be very interesting to study in sheep which immune cells molecular patterns are modulated when 5-HT levels are significantly higher than physiological ones.

## Conclusion

The current study is the first investigation of the effect of toxoplasmosis infection on plasma 5-HT concentrations in one of the most susceptible hosts. These preliminary results can be interesting both for human and animal medicine to determine which neuroendocrine pathways are involved in *T. gondii* infection.

Further studies, supported by more experimental animals, are necessary to understand the role of 5-HT response in infected sheep and to understand if the different degree of susceptibility to infection among animal species may somehow relate to changes of 5-HT circulating levels.

## Authors’ Contributions

AMFM and AMF conceived the idea for the project and designed the experiments. AC performed the experiments. AC and GB analyzed data. RPG had the responsibilities for the sample collection. AMF and AC wrote the manuscript. AMFM and AMF oversaw all stages of the present study and drafted and revised this manuscript. All authors read and approved the final manuscript.
